# Coenzyme Q_10_ partially restores pathological alterations in a macrophage model of Gaucher disease

**DOI:** 10.1186/s13023-017-0574-8

**Published:** 2017-02-06

**Authors:** Mario de la Mata, David Cotán, Manuel Oropesa-Ávila, Marina Villanueva-Paz, Isabel de Lavera, Mónica Álvarez-Córdoba, Raquel Luzón-Hidalgo, Juan M. Suárez-Rivero, Gustavo Tiscornia, José A. Sánchez-Alcázar

**Affiliations:** 1 Centro Andaluz de Biología del Desarrollo (CABD), Consejo Superior de Investigaciones Científicas, Universidad Pablo de Olavide, Carretera de Utrera Km 1, Sevilla, 41013 Spain; 2 Centro de Investigación Biomédica en Red: Enfermedades Raras, Instituto de Salud Carlos III, Madrid, 28029 Spain; 3 Department of Biomedical Sciences and Medicine, University of Algarve, Faro, Portugal

**Keywords:** Gaucher disease, Coenzyme Q_10_, Mitochondria, Oxidative stress, Inflammasome, Efferocytosis

## Abstract

**Background:**

Gaucher disease (GD) is caused by mutations in the GBA1 gene which encodes lysosomal β-glucocerebrosidase (GCase). In GD, partial or complete loss of GCase activity causes the accumulation of the glycolipids glucosylceramide (GlcCer) and glucosylsphingosine in the lysosomes of macrophages.

In this manuscript, we investigated the effects of glycolipids accumulation on lysosomal and mitochondrial function, inflammasome activation and efferocytosis capacity in a THP-1 macrophage model of Gaucher disease. In addition, the beneficial effects of coenzyme Q_10_ (CoQ) supplementation on cellular alterations were evaluated. Chemically-induced Gaucher macrophages were developed by differentiateing THP-1 monocytes to macrophages by treatment with phorbol 12-myristate 13-acetate (PMA) and then inhibiting intracellular GCase with conduritol B-epoxide (CBE), a specific irreversible inhibitor of GCase activity, and supplementing the medium with exogenous GlcCer. This cell model accumulated up to 16-fold more GlcCer compared with control THP-1 cells.

**Results:**

Chemically-induced Gaucher macrophages showed impaired autophagy flux associated with mitochondrial dysfunction and increased oxidative stress, inflammasome activation and impaired efferocytosis. All abnormalities were partially restored by supplementation with CoQ.

**Conclusion:**

These data suggest that targeting mitochondria function and oxidative stress by CoQ can ameliorate the pathological phenotype of Gaucher cells. Chemically-induced Gaucher macrophages provide cellular models that can be used to investigate disease pathogenesis and explore new therapeutics for GD.

**Electronic supplementary material:**

The online version of this article (doi:10.1186/s13023-017-0574-8) contains supplementary material, which is available to authorized users.

## Background

In LSDs, mutations in lysosomal hydrolases or transporters result in the accumulation of specific macromolecules, leading to progressive reduction in the capacity of the lysosome for normal degradation processes, which in turn leads to secondary changes such as impairment in autophagic flux, mitochondrial dysfunction and inflammation [1]. Gaucher disease (GD), the LSD with the highest prevalence, is caused by mutations in the GBA1 gene that results in defective and insufficient activity of the enzyme β-glucocerebrosidase (GCase). Decreased catalytic activity and/or instability of GCase leads to accumulation of glucosylceramide (GlcCer) and glucosylsphingosine in the lysosomes of macrophages. Three clinical forms (phenotypes) of the disease are commonly recognized (Types 1, 2 and 3) of which by far the most severe are those affecting the brain (Types 2 and 3). Current treatments for GD include enzyme replacement therapy with recombinant GCase and substrate-reduction therapy which decreases the biosynthesis of glucosylceramides and thereby reduces their accumulation [2]. Many studies have implicated mitochondrial dysfunction in the pathogenesis of lysosomal diseases in general and in GD in particular [3, 4]. The most pronounced effect occurs in macrophages that participate in ingesting blood cells and apoptotic lymphocytes. Thus, the primary cell type affected in GD is the lipid laden macrophage known as Gaucher cell. As macrophages normally degrade large amounts of cellular membrane lipids by phagocytosis, when GCase is absent or impaired, glycosphingolipids accumulate within the macrophage lysosome and the engorged cells in turn are deposited in the liver, spleen, and lung, causing organ enlargement and progressive dysfunction. GCase is distinguished from other O-glycosyl hydrolases by an acidic pH optimum and a preference for glycolipids. Little is known about the mechanisms by which GlcCer accumulation leads to disease phenotype, particularly for those in which severe neuropathology occurs. Specifically, it is not known if altered macrophage function is responsible for all of the pathological manifestations in all affected tissues, or whether secondary biochemical changes caused directly by GlcCer accumulation in the specific tissues also play a role in the pathological process. Therefore, determining how GlcCer accumulation perturbs the function of lysosomes and other organelles can be important in elucidating the cascade of events that give rise to the pathological consequences in GD.

In order to mimic the pathological phenotype of the disease, an in vitro cellular model of Gaucher disease was developed by treating the THP-1, a human monocytic cell line differentiated into macrophage, with a specific inhibitor of GCase, conduritol beta epoxide (CBE) [5] and the concomitant supplementation with exogenous GlcCer (chemically-induced Gaucher THP-1 macrophages). Autophagy flux, mitochondrial dysfunction, inflammasome activation and efferocitosis capacity were examined in chemically-induced Gaucher THP-1 macrophages. In addition, as mitochondrial dysfunction and/or impaired mitochondria elimination may be associated with alterations of lysosome-dependent processes, treatment with coenzyme Q_10_ (CoQ), an antioxidant and mitochondrial energizer, was evaluated for the improvement of cellular pathological alterations.

## Methods

### Reagents

Monoclonal Anti-Actin and Anti-NLRP3 antibodies were obtained from Sigma-Aldrich (St. Louis, MO). Mitosox Red, Mitotracker Red CMXRos, CMH_2_-DCFDA, 10-N-nonyl acridine orange (NAO), LysoSensor Green DND-189, tetramethylrhodamine methyl ester (TMRM), CellTracker™ Green and Hoechst 33342 were from Invitrogen/Molecular Probes (Eugene, OR). Anti-GCase was obtained from Abcam. Anti-cytochrome c antibody was obtained from BD Biosciences Pharmingen (San Jose, CA) and anti-GAPDH (Glyceraldehyde 3-phosphate dehydrogenase) monoclonal antibody (clone 6 C5) was from Calbiochem-Merck Chemicals Ltd. (Nottingham, UK). CBE, Anti-MAP LC3 (N-20), anti-LAMP-1 were obtained from Santa Cruz Biotechnology (Santa Cruz, CA). Protease inhibitors were from Boehringer Mannheim (Indianapolis, IN). Anti-IL-1β were obtained (Bioss, Inc). Anti-Caspase 1 was obtained from (Cell Signaling Tecnology, CST). The anti-GlcCer rabbit anti-serum was purchased from Glycobiotech GmbH (Kükels, Germany). Glucocerebrosides from Gaucher’s spleen (GlcCer) was obtained from Matreya LCC (Pleasant Gap, PA, USA). The Immun Star HRP substrate kit was from Bio-Rad Laboratories Inc. (Hercules, CA, USA). All other chemicals were purchased from Sigma-Aldrich.

### Chemically-induced Gaucher macrophages

THP-1 cells (human monocytic cell line) were cultured in RPMI medium supplemented with penicillin, streptomycin, and 10% fetal bovine serum at 37 °C in a humidified 5% CO_2_ atmosphere and were first differentiated into macrophages by phorbol 12-myristate 13-acetate (PMA; Sigma-Aldrich) incubation at a final concentration of 100 ng/mL for 3 d and it was followed by 1 d in PMA-free medium before treatments. Then, the Gaucher disease phenotype was induced by chemical inhibition of acid β-glucosidase with 2,5 mM CBE [5]. To exacerbate Gaucher phenotype, the culture medium of THP-1 macrophages was supplemented with exogenous GlcCer (200 μM).

### Immunofluorescence microscopy

Immunofluorescence microscopy was performed using standard methods as previously described [6]. Cover slips were analyzed using a fluorescence microscope (Leica DMRE, Leica Microsystems GmbH, Wetzlar, Germany). Deconvolution studies and 3-dimensional projections were performed using a DeltaVision system (Applied Precision, Issaquah, WA) with an Olympus IX-71microscope.

### Measurement of mitochondrial reactive oxygen species (ROS) production

Mitochondrial ROS generation was assessed using the mitochondrial superoxide indicator MitoSOX Red, according to the manufacturer’s instructions. ROS levels were expressed relative to mitochondrial mass (ROS signal/NAO signal) determined by flow cytometry. Cells were stained with 10 μM NAO for 10 min at 37 °C in the dark.

### Measurement of intracellular H_2_O_2_ content

H_2_O_2_ levels were measured using non fluorescent CMH_2_-DCFDA (5-[and-6]-chloromethyl-2′,7′-dichlorodihydrofluoresceindiacetate, acetyl ester), which diffuses across membranes and is oxidized to fluorescent dichlorofluorescein (DCF). Cultured cells were incubated with CMH_2_-DCFDA diluted in medium at 5 μM for 30 min at 37 °C. After that, cells were analyzed by flow cytometry.

### Determination of mitochondrial membrane potential (ΔΨm)

ΔΨm was measured by staining with 20 nM TMRM or 100 nM Mitotracker Red CMXRos (30 min incubation). Cells were subsequently analyzed by fluorescence microscopy and flow cytometry.

### Immunoblotting analysis

Western blotting was performed using a standard protocol [7] and the Immun Star HRP detection kit (Bio-Rad Laboratories Inc., Hercules, CA, USA).

### Lysosome acidification

Lysosome acidification was measured by staining with 5 μM LysoSensor Green DND-189. LysoSensor was added to cells in growth medium and incubated at 37 °C for 30 min before imaging and flow cytometry analysis. Lysosome acidification was also measured by 10 μg/ml acridine orange staining (15 min incubation at 37 °C). Typically, 10–15 fluorescence microscopy images were collected from 3 separate experiments and the red/green ratio of discrete puncta (*n* = 200) were calculated using Image J.

### Phagocytosis assay

Apoptosis was induced by treatment with 10 μM CPT for 48 h treatment in CellTracker-labelled H460 cells adhered to glass coverslips. Apoptosis was assessed by fluorescence microscopy observing nuclei fragmentation by Hoechst staining, cytochrome c release, and caspase 3 activation. Then, apoptotic cells were co-incubated with control or chemically-induced Gaucher macrophages (150,000 cells/well). After 8 h of co-incubation at 37 °C, cells were fixed in 3.8% paraformaldehyde. The number of control and chemically-induced Gaucher macrophages interacting and engulfing cell fragments was calculated in ten random fields in triplicate by fluorescence microscopy.

### IL-1β levels

Samples from culture media from control and chemically-induced Gaucher macrophages were collected and stored at −80 °C until the assay. IL-1β levels in culture media were determined in triplicates by commercial ELISA kits (Human IL-1β CytoSetTM, Invitrogen, Camarillo, CA, USA).

### Statistical analysis

All results are expressed as mean ± SD of 3 independent experiments. The measurements were statistically analyzed using the Student’s *t* test for comparing 2 groups and analysis of variance for more than 2 groups. The level of significance was set at *p* < 0.05.

## Results

### Establishing a chemically-induced Gaucher macrophage model

**Fig. 1:**
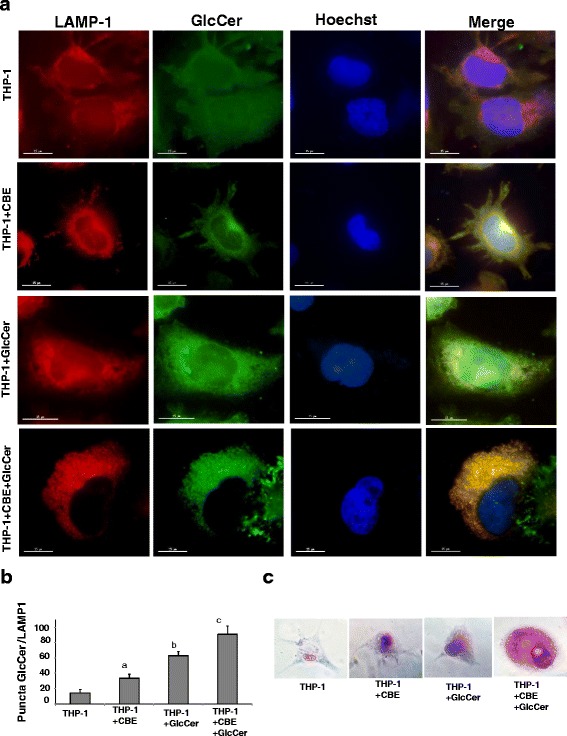
Establishing a chemically-induced Gaucher macrophage model. **a** THP-1 macrophages were cultured in the presence or absence of CBE (2,5 mM), GlcCer (200 μM) and CBE + GlcCer (2,5 mM + 200 μM) for 72 h. Cells were fixed and immunostained with anti-GlcCer and anti-LAMP-1 (Lysosomal marker) and examined by fluorescence microscopy. Lysosomal marker, LAMP-1, or GlcCer were visualized as red or green, respectively. Colocalization of GlcCer signal with LAMP1 indicates GlcCer lysosomal accumulation **b** Quantification of GlcCer/LAMP-1 puncta in control and macrophages incubated with CBE, GlcCer and CBE + GlcCer (*n* = 100 cells). Data represent the mean ± SD of three separate experiments. ^a^ *p* < 0.05 between CBE treatment and control cells. ^b^ *p* < 0.05 between GlcCer supplementation and control cells. ^c^ *p* < 0.05 between CBE + GlcCer combined treatment and CBE or GlcCer treatment. **c** Representative images of Hematoxylin and eosin staining of chemically-induced Gaucher macrophages

First, we examined whether chemically-induced Gaucher THP-1 macrophages reproduce the pathological phenotype of this disease. As shown in Fig. 1a and b, GlcCer accumulated in macrophages treated with CBE for 72 h. This accumulation was significantly increased by exogenous 200 μM GlcCer supplementation (Fig. 1a and b). GlcCer accumulation mainly colocalized with lysosomes which were labeled with LAMP-1 (Fig. 1a and b). Hematoxylin/eosin staining of chemically-induced Gaucher THP-1 macrophages also showed cells with dilated vesicles presumably representing GlcCer accumulation in lysosomes (Fig. 1c). These alterations were particularly evident in cells treated with CBE and supplemented with GlcCer.

As GlcCer accumulation was significantly higher in THP-1 cells treated with CBE and supplemented with GlcCer we decided to work with this model in successive experiments. Appropriate controls showing that pathophysiological alterations are also more pronounced with the combined treatment are provided in (Additional files 1: Figures S1–S8).

### CoQ treatment partially ameliorates GlcCer accumulation in chemically-induced Gaucher THP-1 macrophages

**Fig. 2:**
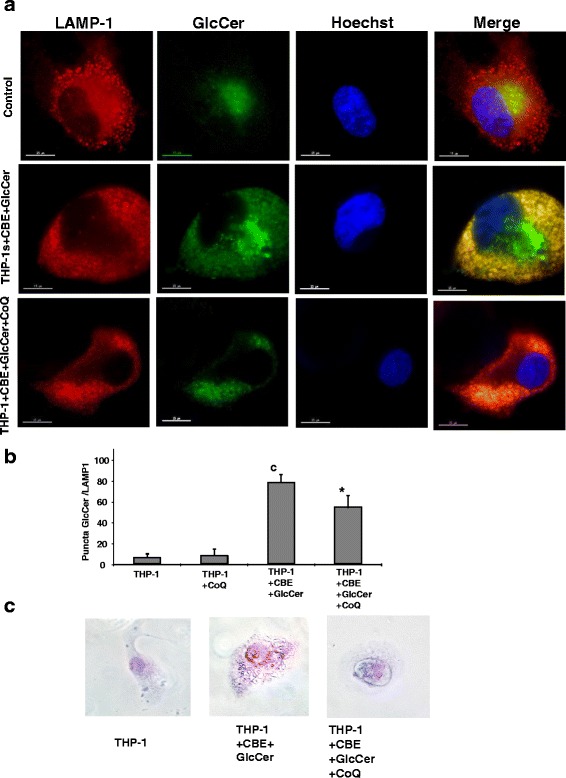
Elevation of GlcCer levels and lysosomal markers colocalization in chemically-induced Gaucher macrophages. **a** Representative images of GlcCer and lysosomal marker LAMP-1 in chemically-induced Gaucher macrophages. THP-1 macrophages were cultured in the presence or absence of CBE + GlcCer (2,5 mM + 200 μM), or CBE + GlcCer + CoQ (2,5 mM + 200 μM + 25 μM) for 72 h. Cells were fixed and immunostained with anti-GlcCer and anti-LAMP-1 (Lysosomal marker) and examined by fluorescence microscopy. **b** Quantification Image analysis of GlcCer/LAMP-1 puncta in chemically-induced Gaucher macrophages incubated with or without CBE + GlcCer and CBE + GlcCer + CoQ (*n* = 100 cells). Data represent the mean ± SD of three separate experiments. ^c^ *p* < 0.05 between control and chemically-induced Gaucher macrophages. *p < 0.05 between the presence and the absence of CoQ treatment. **c** Representative images of Hematoxylin and eosin staining of control, chemically-induced Gaucher macrophages and chemically-induced Gaucher macrophages supplemented with CoQ

As mitochondrial dysfunction has been associated with alterations of lysosome-dependent processes, treatment with CoQ, an antioxidant and mitochondrial energizer, was evaluated for improving glycolipids accumulation in cells treat with CBE and supplemented with ClcCer. Supplementation with CoQ (25 μM) of chemically-induced Gaucher THP-1 macrophages partially reduced GlcCer accumulation and the number of GlcCer/LAMP-1 puncta (Fig. 2a and b). Furthermore, in concordance with this, there was a drastically reduction of dilated vesicles in hematoxylin/eosin stainings (Fig. 2c).

### Autophagic flux is impaired in chemically-induced Gaucher THP-1 macrophage model

**Fig. 3:**
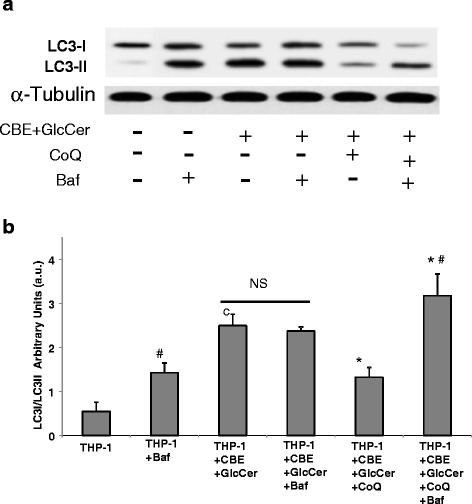
Impaired autophagic flux in chemically-induced Gaucher macrophages. **a** Autophagy flux. Determination of LC3-II expression levels in the presence and absence of bafilomycin A1 in control and chemically-induced Gaucher macrophages. Control and chemically-induced Gaucher macrophages were incubated with bafilomycin A1 (100 nM for 12 h). Total cellular extracts were analyzed by immunoblotting with antibodies against LC3. Alpha-tubulin was used as a loading control. **b** Densitometry of Western blotting was performed using the ImageJ software. Data represent the mean ± SD of three separate experiments. ^c^ *p* < 0.05 between control and chemically-induced Gaucher macrophages. *p < 0.05 between the presence and the absence of CoQ. #*p* < 0.05 between the presence and the absence of bafilomycin A1

As GlcCer accumulation in lysosomes may interfere with lysosomal function and impair lysosomal fusion with autophagosomes, we next examined autophagosome maturation. To ascertain whether or not autophagic flux was impaired in chemically-induced Gaucher THP-1 macrophages, we checked the levels of LC3-II in the presence of bafilomycin A1 (Baf), a specific inhibitor of vacuolar H^+^-ATPases and a blocker of autophagosome-lysosome fusion (Fig. 3a and b). As expected, Baf treatment in control THP-1 macrophages led to a significant increase in the amount of LC3-II suggesting that autophagic flux was normal. However, basal LC3-II levels were increased in chemically-induced Gaucher THP-1 macrophages, suggesting autophagosome accumulation. Furthermore, Baf treatment had no effect in LC3-II levels indicating that autophagic flux was impaired (Fig. 3a and b). Supplementation with CoQ decreased the amount of basal levels of LC3-II. Furthermore, LC3-II expression levels were significantly increased after Baf treatment, suggesting improvement of autophagic flux (Fig. 3a and b).

### Effect of CoQ supplementation on lysosomal pH and mitochondrial membrane potential (ΔΨm) in chemically-induced Gaucher THP-1 macrophages

Little is known about how GlcCer accumulation in lysosomes leads to cellular pathology. One critical question is whether GlcCer mediates all of its pathological effects from within the lysosome, or whether some GlcCer interact with biochemical and cellular pathways located in other organelles as mitochondria.

**Fig. 4:**
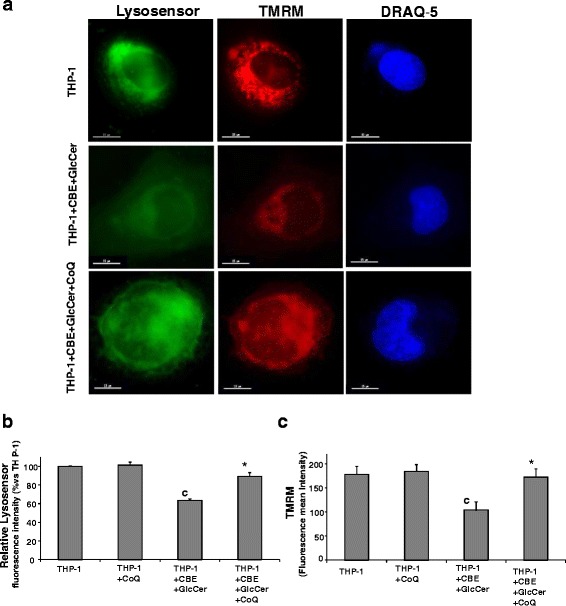
Increase lysosomal pH is associated with decreased mitochondrial membrane potential (ΔΨm) in chemically-induced Gaucher macrophage. **a** Representative images of control and chemically-induced Gaucher macrophages stained with LysoSensor Green DND-189 which accumulates in acidic organelles and exhibits green fluorescence, and TMRM, a potentiometric fluorescent indicator that exhibits red fluorescence in mitochondria. Effect of CoQ (25 μM) supplementation for 72 h on lysosomal pH and ΔΨm in chemically-induced Gaucher macrophages. **b** Determination of lysosomal pH in both control and chemically-induced Gaucher macrophages by staining with LysoSensor Green DND-189 coupled to flow cytometry analysis. Data represent the mean ± SD of three separate experiments. ^c^ *p* < 0.05 between control and chemically-induced Gaucher macrophages. *p < 0.05 between the presence and the absence of CoQ treatment. **c** Determination ΔΨm was assessed by TMRM staining and flow cytometry analysis. Data represent the mean ± SD of three separate experiments. ^c^ *p* < 0.05 between control and chemically-induced Gaucher macrophages. *p < 0.05 between the presence and the absence of CoQ treatment

As in Gaucher’s disease the accumulation of GlcCer has been associated with an elevation in lysosomal pH [8], we first determined whether GlcCer accumulation in chemically-induced Gaucher THP-1 macrophages affects lysosomal pH. Results indicated that the accumulation of GlcCer impaired the acidification of these vesicles (Fig. 4a). Flow cytometry analysis confirmed that LysoSensor Green DND-189 fluorescence was decreased in chemically-induced Gaucher THP-1 macrophages (Fig. 4b). In addition, to assess mitochondrial dysfunction in chemically-induced Gaucher THP-1 macrophages, ΔΨm was evaluated by TMRM staining and fluorescence microscopy visualization. TMRM fluorescence was decreased in chemically-induced Gaucher THP-1 macrophages, which reflects mitochondrial depolarization (Fig. 4a). Mitochondrial depolarization was also confirmed by flow cytometry analysis (Fig. 4c).

To elucidate whether CoQ had a beneficial effect on lysosomal pH and ΔΨm impairment, chemically-induced Gaucher macrophages were treated with 25 μM CoQ for 72 h. CoQ treatment resulted in a significant improvement of both lysosomal pH and ΔΨm (Fig. 4a, b and c).

**Fig. 5:**
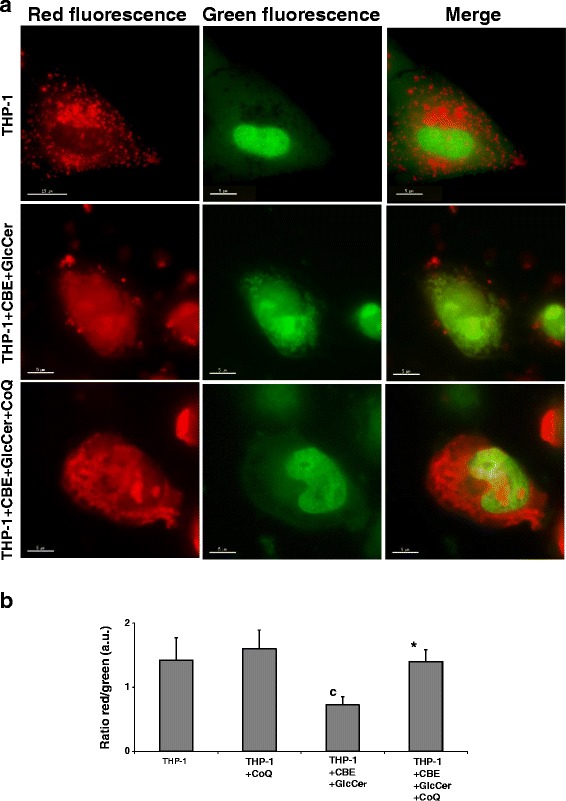
Lysosome acidification impairment in chemically-induced Gaucher macrophages. **a** Representative fluorescence images of THP-1 macrophages cultured for 72 h with CBE + GlcCer (2,5 mM + 200 μM) in the presence of CoQ (25 μM), and stained for 15 min with 10 μg/ml acridine orange. **b** Quantification of the ratio between the red and green signal of acridine orange was performed by immunofluorescence microscopy using the Image J software. Chemically-induced Gaucher THP-1 macrophages showed reduced red fluorescence and increased green fluorescence and a notably reduction in the red/green signal ratio suggesting decreased lysosomal acidity. Data represent the mean ± SD of three separate experiments. Quantification of the ratio between the red and green signal of acridine orange was performed by immunofluorescence microscopy using the Image J software (*n* = 100 cells). ^c^ *p* < 0.05 between control and chemically-induced Gaucher macrophages. *p < 0.05 between the presence and the absence of CoQ treatment

Lysosome acidification impairment in chemically-induced Gaucher macrophages was also confirmed by acridine orange staining. Chemically-induced Gaucher macrophages showed a decrease in the red/green ratio after acridine orange staining consistent with decreased lysosomal acidity (Fig. 5a and b). Supplementation with CoQ (25 μM) significantly increased the red/green ratio.

### Effect of CoQ on reactive oxygen species (ROS) production in chemically-induced Gaucher macrophages

**Fig. 6:**
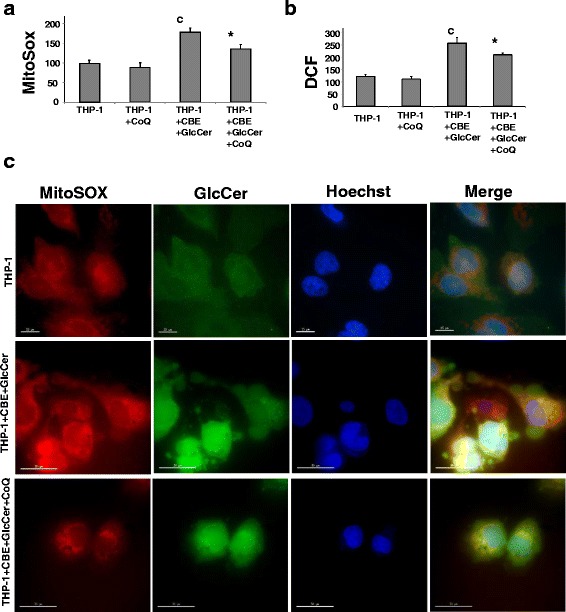
Increase ROS production in chemically-induced Gaucher macrophage. **a** Mitochondrial ROS levels in control and chemically-induced Gaucher macrophages. Results are expressed as the ratio of MitoSOX signal to 10-N-nonyl acridine orange signal in the absence or presence of CoQ (25 μ M) for 72 h. MitoSOX and 10-N-nonyl acridine orange signal were determined by flow cytometry analysis. Data represent the mean ± SD of three separate experiments. ^c^ *p* < 0.05 between control and chemically-induced Gaucher macrophages. *p < 0.05 between the presence and the absence of CoQ treatment. **b** H_2_O_2_ levels in control and chemically-induced Gaucher macrophages by CMH2-DCFDA staining coupled with flow cytometry analysis. H_2_O_2_ levels in control and chemically-induced Gaucher macrophages cultured in the absence or presence of CoQ (25 μ M) for 72 h. Data represent the mean ± SD of three separate experiments. ^c^ *p* < 0.05 between control and chemically-induced Gaucher macrophages. *p < 0.05 between the presence and the absence of CoQ treatment. **c** Representative fluorescence images of MitoSox (*red*) and GlcCer (*green*) staining

It is well established that mitochondrial dysfunction is associated with increased ROS production [9]. Therefore, we examined mitochondrial ROS and H_2_O_2_ levels in chemically-induced Gaucher macrophages. Mitochondrial superoxide production and H_2_O_2_ levels were increased approximately by 2,5-fold and by 2-fold respectively (Fig. 6a and b), suggesting increased oxidative stress in chemically-induced Gaucher macrophages. Supplementation with CoQ (25 μM), induced a notably reduction in mitochondrial superoxide and H_2_O_2_ levels in chemically-induced Gaucher macrophages, but had no effect in control cultures (Fig. 6a and b).

### Effect of CoQ on mitophagy in chemically-induced Gaucher macrophages

**Fig. 7:**
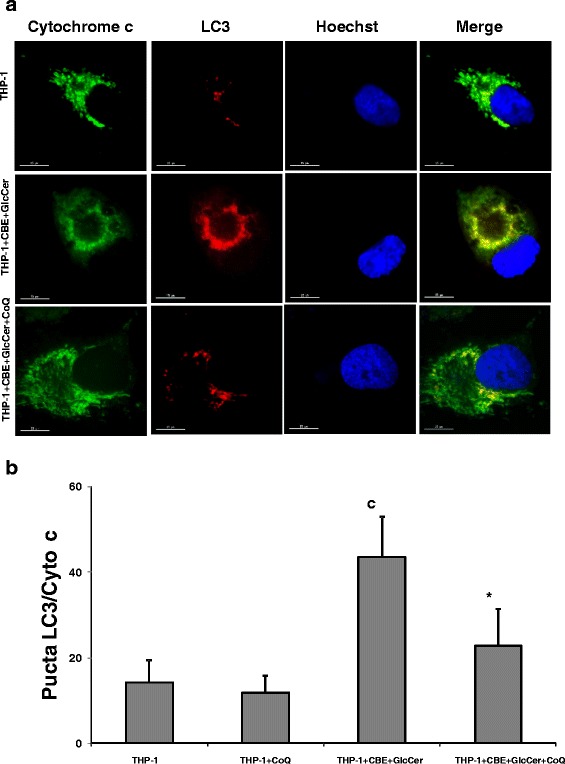
Colocalization of autophagosome and mitochondria markers in chemically-induced Gaucher macrophages. **a** Mitochondria Image analysis of LC3 and cytochrome c immunostaining in control and chemically-induced Gaucher macrophages. Control and chemically-induced Gaucher macrophages were cultured in the presence or absence of CoQ (25 μM) for 72 h. Cells were fixed and immunostained with anti-LC3 (autophagosome marker) and cytochrome c (mitochondrial marker) and examined by fluorescence microscopy. **b** Quantification of LC3/cytochrome c puncta in control and chemically-induced Gaucher macrophages incubated with or without CoQ (*n* = 100 cells). Data represent the mean ± SD of three separate experiments. ^c^ *p* < 0.05 between control and chemically-induced Gaucher macrophages. *p < 0.05 between the presence and the absence of CoQ treatment

Mitochondria can be degraded through mitophagy. To determine whether the accumulated autophagosomes in chemically-induced Gaucher macrophages contained mitochondria, we performed immunofluorescence double staining with antibodies against LC3 (autophagosome marker) and cytochrome c (mitochondrial marker) (Fig. 7a). LC3 staining was markedly increased in chemically-induced Gaucher THP-1 macrophages respect to control macrophages. In addition, LC3 signal strongly colocalized with cytochrome c, suggesting that mitochondria are engulfed by autophagosomes in chemically-induced Gaucher THP-1 macrophages. Supplementation with CoQ (25 μM) partially reduced the number of LC3/cytochrome c puncta (Fig. 7b).

### Effect of CoQ on inflammasome activation in chemically-induced Gaucher macrophages

**Fig. 8:**
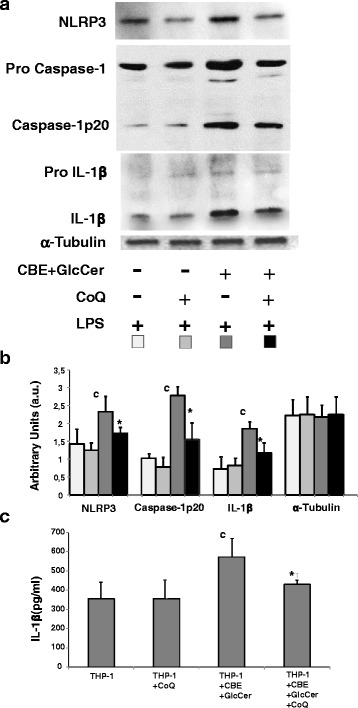
Inflammasome activation in chemically-induced Gaucher macrophages. **a** Western blot analysis of NLRP3, caspase-1 and IL-1β in control and chemically-induced Gaucher macrophages treated with CoQ (25 μM) for 72 h. Cells were supplemented with lipopolysaccharides (LPS) the last 24 h. Alpha-tubulin was used as loading control. **b** Densitometric analysis of Western blottings. Data represent the mean ± SD of three separate experiments. ^c^ *p* < 0.05 between control and chemically-induced Gaucher macrophages. *p < 0.05 between the presence and the absence of CoQ treatment. **c** IL-1β levels were determined by ELISA assay as described in Material and Methods. Data represent the mean ± SD of three separate experiments. ^c^ *p* < 0.05 between control and chemically-induced Gaucher macrophages. *p < 0.05 between the presence and the absence of CoQ treatment

To determine the effect of GlcCer accumulation on inflammasome activation, we evaluated NLRP3 expression levels and caspase-1 activation in chemically-induced Gaucher macrophages. We found increased NLRP3 expression levels and caspase-1 cleavage as well as enhanced levels of intracellular and secreted IL-1β compared to controls (Fig. 8a, b and c). CoQ treatment resulted in a significant decrease in NLRP3 expression levels, caspase-1 cleavage and intracellular and secreted IL-1β levels (Fig. 8a, b and c).

### Defective efferocytosis in chemically-induced Gaucher macrophages

**Fig. 9:**
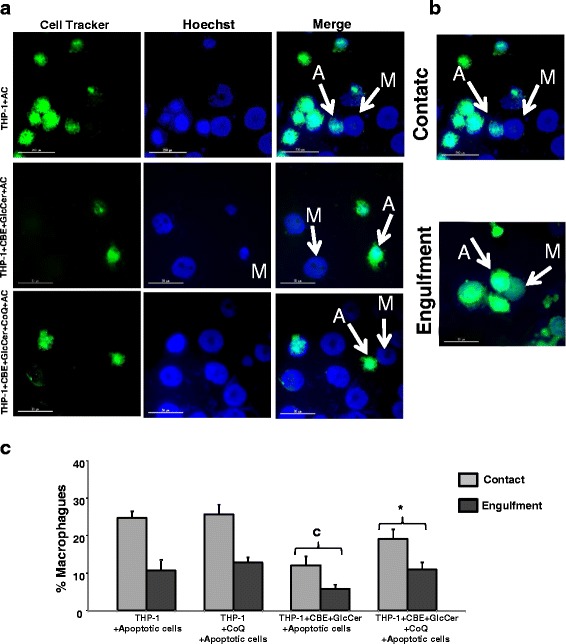
Defective efferocytosis in chemically-induced Gaucher macrophages. **a** Representative fluorescence images of CellTracker™ Green-labelled control and apoptotic H460 cells interacting with control and chemically-induced Gaucher macrophages (M). Nuclear morphology was revealed by staining with Hoechst 33342 (1 μg/ml). **b** Representative images of macrophages during contact and engulfment of apoptotic cells. **c** Proportion of chemically-induced Gaucher macrophages interacting and engulfing of apoptotic cells treated with CoQ (25 μM) for 72 h. Data represent the mean ± SD of three separate experiments. ^c^ *p* < 0.05 between control and chemically-induced Gaucher macrophages. *p < 0.05 between the presence and the absence of CoQ treatment

Given that apoptotic cells are rapidly phagocytosed by macrophages, a process that represents a critical step in tissue remodeling, immune responses, and the resolution of inflammation, we evaluated the phagocytosis capacity of chemically-induced Gaucher macrophages. In vitro phagocytosis assays indicate a defective efferocytosis by chemically-induced Gaucher macrophages with a significant decrease of contacts and engulfment of apoptotic cells. CoQ treatment resulted in a significant increase of efferocytosis capacity in chemically-induced Gaucher macrophages (Fig. 9a, b and c).

## Discussion

Our study shows that GCase deficient activity and accumulation of GlcCer in a macrophage model of GD can cause lysosomal and mitochondrial dysfunction associated with inflammasome activation and impaired efferocytosis. This study also showed that it is possible to ameliorate the cellular pathological consequences of GlcCer accumulation by targeting mitochondria and oxidative stress with CoQ treatment.

In order to mimic the disease state, an in vitro model of Gaucher disease was developed by treating THP-1macrophagues with a specific irreversible inhibitor of GCase, CBE, and exogenous GlcCer supplementation. In previous works, GCase deficiency has been mimicked treating he human neuroblastoma SHSY-5Y cell line with CBE [10]. The treatment with CBE resulted in fragmentation of mitochondria, significant progressive decline in mitochondrial membrane potential, reduction of ATP synthesis and an increase in ROS production. Furthermore, an animal model and in vitro models for Gaucher disease have been produced by injecting mice or treating macrophages with CBE, causing intracellular storage of endogenous GlcCer [11, 12]. However, in addition to endogenously synthesized GlcCer, storage material in Gaucher cells is also thought to originate from the turnover of exogenously derived lipids in cell membranes of phagocytosed red and white blood cells. For this reason and in order to exacerbate the disease phenotype, in addition to GCase inhibition, we supplemented the culture medium with exogenous GlcCer. In our cell model, lipid storage would be expected to occur much more rapidly and to more closely mimic the disease state. These GlcCer-laden Gaucher macrophages have a characteristic morphology with extensive presence of lysosomal lipid deposits. Currently, many of the available mouse models of Gaucher disease are not suitable for these studies because most knock-in mouse models carrying human mutations in glucocerebrosidase do not display an accurate disease phenotype or are lethal [13]. Thus, cell-based Gaucher disease models may provide an alternative approach for evaluating the efficacy of new therapeutic strategies.

In GD, accumulation of sphingolipids has been shown to alter autophagy by reducing autophagosome clearance, and so promoting their accumulation [14]. Indeed, alteration of autophagic flux has been demonstrated in GD cell models [3]. Furthermore, increased number of autophagosomes has been observed in hypomorphic prosaposin mice carrying the homozygous V394L Gba1 mutation that showed accumulation of GlcCer [15].

Degradation of engulfed material is primarily mediated by lysosomal enzymes that function optimally within a narrow range of acidic pH values. Elevation of lysosomal pH in Gaucher cells interferes with the degradation process and may contribute to the associated pathologies [16]. Recently, there have been increased reports showing that lysosomal pH may be regulated [17] and that GlcCer accumulation may have an important role in its dysregulation [8]. Our results showed that GlcCer accumulation impaired lysosome acidification and as a result may alter the activity of lysosomal hydrolases which may result in secondary substrate accumulation [18]. The accumulation of primary and secondary substrates provokes a cascade of events that impacts not only the endosomal–autophagic–lysosomal system, but also in other organelles including mitochondria, the ER, Golgi, peroxisomes, and overall the cell function [1].

Furthermore, our results confirm previous experiments that showed that autophagic flux is reduced in most LSDs [19]. This is evident from the combined elevation of autophagic substrates and autophagosome-associated LC3-II in chemically-induced Gaucher macrophages.

Constitutive macroautophagy maintains mitochondrial quality by selectively degrading dysfunctional mitochondria via a process known as mitophagy [20]. Therefore, reduced autophagic flux in LSDs may lead to the persistence of dysfunctional mitochondria [21–25]. In addition of impaired mitochondria quality control, some authors have hypothesized that variations in GlcCer and ceramide might play an important role in the development of mitochondrial dysfunction in GD [26]. Furthermore, it has been reported that the sphingolipid ceramides provoke oxidative stress by disrupting mitochondria and inducing lethal mitophagy [27]. In agreement with these results, we have previously reported that GlcCer is accumulated mainly in the lysosomal and mitochondrial compartments in fibroblasts derived from Gaucher patients and that both accumulation of GlcCer and impairment of autophagic flux may induce mitochondrial dysfunction in Gaucher disease [3].

Dysfunctional mitochondria are involved in the pathogenesis of several neurodegenerative diseases. The proper elimination of damaged mitochondria is needed in post-mitotic neurons because progressive accumulation of damaged mitochondria might eventually lead to cell death. Mitochondrial dysfunction with reduced respiratory chain complex activities, increased ROS production and decreased potential in neurons and astrocytes has recently been reported in a mouse model of type II neuronopathic GD [28].

GlcCer accumulation within inflammatory cells as macrophages may contribute to persistent and altered inflammatory responses in GD. In GD patients, elevated levels of some cytokines and chemokines have been reported including IL-1β, interleukin-1 receptor antagonist, IL-6, IL-8, IL-10, IL18, TNF-α, M-CSF, and pulmonary and activation-regulated chemokine (PARC or CCL-18) [29–32]. A similar finding was noted using THP-1 cells differentiated into macrophages by retinoic acid and treated with the GCase inhibitor CBE [33]. Normal human mesenchymal stromal cells treated with CBE also showed an up-regulation of genes involved in proteolysis, lipid homeostasis, and the inflammatory response [34]. In this manuscript, we show that impaired autophagic flux is associated with inflammasome activation and increased maturation of IL-1β in a chemically-induced Gaucher macrophage model. These findings provide a link between impaired autophagy and increased secretion of pro-inflammatory cytokines in Gaucher cells. Consistent with our results, macrophages derived from peripheral monocytes from patients with type 1 Gaucher disease with genotype N370S/N370S showed an increased secretion of interleukins IL-1β and IL-6 [35]. Our findings are also supported by studies in patients with type 1 GD [29], in mouse models of GD [36] and in iPSC‐derived cells [37].

However the exact mechanism by which GlcCer accumulation activates the NLRP3 inflammasome is not yet understood. Several recent data have shown that autophagy, and in particular mitophagy, are key links among inflammasome, ROS production and mitochondrial dysfunction [38, 39] .

Macrophages are involved in many essential processes including the removal of pathogens and dead cells through phagocytosis [40]. This may contribute to the accumulation of unphagocytosed debris from cells undergoing apoptosis in the course of homeostatic tissue remodeling and repair. Mutant GBA macrophages accumulate undigested lysosomal material, which disrupts endocytic recycling and impairs their migration and engulfment of dying cells. This causes a buildup of unengulfed cell debris.

Given that chemically-induced Gaucher macrophages manifest their defective storage phenotype, we also evaluated their phagocytosis capability. We found impaired efferocytosis in chemically-induced Gaucher macrophages. In agreement with our findings, impaired microbicidal capacity of mononuclear phagocytes from patients with type I Gaucher Disease has been previously reported [41].

Given that defects in energy metabolism and oxidative stress have been demonstrated to play a role in the pathogenesis of GD, we envisioned that the treatment with coenzyme CoQ could also exert beneficial therapeutic effects. The fundamental role of CoQ in mitochondrial bioenergetics and its well-acknowledged antioxidant properties constitute the basis for its clinical applications, although some of its effects may be related to a gene induction mechanism [42]. Interestingly for the treatment of neuropathic GD, CoQ is also able to cross the BBB [43].

The treatment with CoQ is currently considered as a potential experimental drug for the treatment of neurodegenerative diseases in general [44] and lysosomal diseases in particular [45]. Apart from mitochondria where CoQ functions as an electron and proton donor in the mitochondrial respiratory chain, high levels of CoQ have been also reported in lysosomes. CoQ plays a key role in the exchange of electrons in lysosomal membrane, which contributes to protons’ translocation into the lumen and to the acidification of intra-lysosomal medium, which is essential for the proteolytic function of hydrolases responsible–when deficient- of a wide range of inherited lysosomal diseases [46]. Consistent with these findings, the treatment with CoQ improved mitochondrial/lysosomal function, increased autophagic flux and reduced inflammasome activation as well as improved efferocytosis capacity of chemically-induced Gaucher macrophages.

These results, however, should be interpreted with caution since the positive effects of CoQ in vitro may not have an equivalent beneficial effect when translated to human clinical trials as it has been recently demonstrated in two large trials in Parkinson and Huntington diseases [47, 48].

## Conclusion

Our results support the hypothesis that lysosomal dysfunction interferes with the clearance of damaged mitochondria and that the two critical pathways, lysosomal and mitochondrial dysfunction converge in the pathogenesis of GD. In addition, CoQ supplementation partially corrected many of the cellular pathophysiological alterations. Therefore, we proposed that boosting lysosomal function in conjunction with improvements of mitochondrial function will have a protective effect on GD. Studies in a suitable animal model may provide preclinical data, which may support clinical trials with CoQ given in human patients with GD.

## Additional file

Additional file 1: Supplementary data. (PDF 2022 kb)

## Abbreviations

AO: Acridine orange
BAF: Bafilomycin A1
BBB: Blood brain barrier
CBE: Conduritol B-epoxide
CMH2-DCFDA: (5-[and-6]-chloromethyl-2′,7′-dichlorodihydrofluoresceindiacetate, acetyl ester)
CNS: Central nervous system
CoQ: Coenzyme Q_10_
ER: Endoplasmic reticulum
ERAD: ER associated degradation system
ERT: Enzyme replacement therapy
GCase: β-glucocerebrosidase
GD: Gaucher disease
GlcCer: Glucosylceramide
GlcSph: Glucosylsphingosine
IgGs: Immunoglobulins G
LPS: Lipopolysaccharide
LSDs: Lysosomal storage diseases
PD: Parkinson’s disease
PMA: Phorbol 12-myristate 13-acetate
ROS: Reactive oxygen species
SRT: Small-molecule substrate reduction therapy
ΔΨm: mitochondrial membrane potential
