# Supplementary material for: Coenzyme Q10 partially restores pathological alterations in a macrophage model of Gaucher disease
Source: Orphanet J Rare Dis. 2017 Feb 6;12:23. doi: 10.1186/s13023-017-0574-8 (PMC5292786; doi:10.1186/s13023-017-0574-8)
Supplement: Additional file 1: — Supplementary data. (PDF 2022 kb) [file 13023_2017_574_MOESM1_ESM.pdf]

Supplementary Figures (S1-8)

Figure legends

Figure S1. Increased lysosomal pH is associated with decreased mitochondrial membrane potential (ΔΨm) in chemically-induced Gaucher macrophages. a) Representative images of control and chemically-induced Gaucher macrophages stained with LysoSensor Green DND-189 and TMRM. THP-1 macrophages were cultured in the presence or absence of CBE (2,5mM), GlcCer (200 µM) and CBE+GlcCer (2,5mM+200µM) for 72 h. Effect of CBE, GlcCer and CBE+GlcCer supplementation for 72 h on Lysosomal pH and ΔΨm in chemically-induced Gaucher macrophages. **b)** Determination of Lysosomal pH in both control and chemically-induced Gaucher macrophages by staining with LysoSensor Green DND-189 coupled to flow cytometry analysis. **c)** Determination ΔΨm was assessed by TMRM staining and flow cytometry analysis. Data represent the mean±SD of three separate experiments. $^{a}p<0.05$ between CBE treatment and control cells. $^{b}p<0.05$ between GlcCer supplementation and control cells. $^{c}p<0.05$ between CBE+GlcCer combined treatment and CBE or GlcCer treatment.

Figure S2. **Lysosome acidification impairment in chemically-induced Gaucher macrophages. a)** Representative images of chemically-induced Gaucher macrophages stained with acridine orange. THP-1 macrophages were cultured in the presence or absence of CBE (2,5mM), GlcCer (200 µM) and CBE+GlcCer (2,5mM+200µM) for 72 h, and stained for 15 min with 10 µg/ml acridine orange. **b)** Quantification of the ratio between the red and green signal of acridine orange. Chemically-induced Gaucher THP-1 macrophages showed reduced red fluorescence and increased green fluorescence and a notably reduction in the red/green signal ratio suggesting decreased lysosomal acidity. Data represent the mean±SD of three separate experiments. Quantification of the ratio

between the red and green signal of acridine orange was performed by immunofluorescence microscopy in control and macrophages incubated with CBE, GlcCer and CBE+GlcCer using the Image J software (n = 100 cells). Data represent the mean±SD of three separate experiments. [a]$p < 0.05$ between CBE treatment and control cells. [b]$p<0.05$ between GlcCer supplementation and control cells. [c]$p<0.05$ between CBE+GlcCer combined treatment and CBE or GlcCer treatment.

Figure S3. Impaired autophagic flux in chemically-induced Gaucher macrophages.
a) Determination of LC3-II expression levels in the presence and absence of bafilomycin A1. Control and THP-1 macrophages were cultured in the presence or absence of CBE (2,5mM), GlcCer (200 μM) and CBE+GlcCer (2,5mM+200μM) for 72 h. Control and chemically-induced Gaucher macrophages were incubated with bafilomycin A1 (100 nM for 12 h). Total cellular extracts were analyzed by immunoblotting with antibodies against LC3. α-Tubulin was used as a loading control. **b)** Densitometry of Western blotting was performed using the ImageJ software. Data represent the mean±SD of three separate experiments. [a]$p < 0.05$ between CBE treatment and control cells. [b]$p<0.05$ between GlcCer supplementation and control cells. [c]$p<0.05$ between CBE+GlcCer combined treatment and CBE or GlcCer treatment. #$p<0.05$ between the presence and the absence of bafilomycin A1.

Figure S4. Increase ROS production in chemically-induced Gaucher macrophages.
a) Mitochondrial ROS levels in control and chemically-induced Gaucher macrophages by treatment with CBE (2,5mM), GlcCer (200 μM) and CBE+GlcCer (2,5mM+200μM) for 72 h. Results are expressed as the ratio of MitoSOX signal to 10-N-nonyl acridine orange signal. MitoSOX and 10-N-nonyl acridine orange signal were determined by

flow cytometry analysis. **b)** H_2O_2 levels in control and chemically-induced Gaucher macrophages by CMH2-DCFDA staining coupled with flow cytometry analysis. H_2O_2 levels in control and chemically-induced THP-1 macrophages were cultured in the absence or presence of CBE (2,5mM), GlcCer (200 μM) and CBE+GlcCer (2,5mM+200μM) for 72 h. Data represent the mean±SD of three separate experiments. $^a p<0.05$ between CBE treatment and control cells. $^b p<0.05$ between GlcCer supplementation and control cells. $^c p<0.05$ between CBE+GlcCer combined treatment and CBE or GlcCer treatment. **c)** Representative fluorescence images of MitoSox(red) and GlcCer (green) stained cells.

Figure S5. Inflammasome activation in chemically-induced Gaucher macrophages. **a)** Western blot analysis of NLRP3, caspase-1 and IL-1β in control and chemically-induced. THP-1 macrophages were cultured in the absence or presence of CBE (2,5mM), GlcCer (200 μM) and CBE+GlcCer (2,5mM+200μM) for 72 h. Cells were supplemented with Lipopolysaccharides (LPS) the last 24 hours. Alpha-tubulin was used as loading control. **b)** Densitometric analysis of Western blottings. Data represent the mean±SD of three separate experiments. $^a p<0.05$ between CBE treatment and control cells. $^b p<0.05$ between GlcCer supplementation and control cells. $^c p<0.05$ between CBE+GlcCer combined treatment and CBE or GlcCer treatment. **c)** IL-1β levels were determined by ELISA assay as described in Material and Methods. Data represent the mean±SD of three separate experiments. $^a p < 0.05$ between CBE treatment and control cells. $^b p<0.05$ between GlcCer supplementation and control cells. $^c p<0.05$ between CBE+GlcCer combined treatment and CBE or GlcCer treatment.

Figure S6. Mitochondrial membrane potential (ΔΨm) in chemically-induced Gaucher macrophages. a) Determination ΔΨm was assessed by MitoTracker staining and flow cytometry analysis. THP-1 macrophages were cultured in the absence or presence of CBE (2,5mM), GlcCer (200 μM) and CBE+GlcCer (2,5mM+200μM) for 72 h. Data represent the mean±SD of three separate experiments. [a]p<0.05 between CBE treatment and control cells. [b]p<0.05 between GlcCer supplementation and control cells. [c]p<0.05 between CBE+GlcCer combined treatment and CBE or GlcCer treatment.
b) Representative images of MitoTracker and cytochrome c staining in control and chemically-induced Gaucher macrophages cultured in the absence or presence of CBE (2,5mM), GlcCer (200 μM) and CBE+GlcCer (2,5mM+200μM) for 72 h.

Figure S7. Effect of GlcCer accumulation on mitochondrial function in chemically-induced Gaucher macrophages. a) Quantification of GlcCer/Mitochondria puncta in control and THP-1macrophages incubated with CBE, GlcCer and CBE+GlcCer (n = 100 cells). Data represent the mean±SD of three separate experiments. [a]p < 0.05 between CBE treatment and control cells. [b]p<0.05 between GlcCer supplementation and control cells. [c]p<0.05 between CBE+GlcCer combined treatment and CBE or GlcCer treatment. **b)** Representative images of Mitotracker and anti-GlcCer anti-sera staining in control and THP-1 macrophages were cultured in the presence or absence of CBE (2,5mM), GlcCer (200 μM) and CBE+GlcCer (2,5mM+200μM) for 72 h. Cells were fixed and immunostained with anti-GlcCer and MitoTracker and examined by fluorescence microscopy. Mitochondrial marker, MitoTracker, or GlcCer were visualized as red or green, respectively. Colocalization of GlcCer signal with MitoTracker indicates GlcCer mitochondrial accumulation.

Figure S8. Defective efferocytosis in chemically-induced Gaucher macrophages. a) Representative fluorescence images of CellTracker™ Green-labelled control and apoptotic H460 cells (A) interacting with control and chemically-induced Gaucher macrophages (M). THP-1 macrophages were cultured in the absence or presence of CBE (2,5mM), GlcCer (200 μM) and CBE+GlcCer (2,5mM+200μM) for 72 h (M). Nuclear morphology was revealed by staining with Hoechst 33342 (1μg/ml). **b)** Proportion of chemically-induced Gaucher macrophages interacting and engulfing of apoptotic cells. THP-1 macrophages were cultured in the absence or presence of CBE (2,5mM), GlcCer (200 μM) and CBE+GlcCer (2,5mM+200μM) for 72 h. Data represent the mean±SD of three separate experiments. $^{a}p<0.05$ between CBE treatment and control cells. $^{b}p<0.05$ between GlcCer supplementation and control cells. $^{c}p<0.05$ between CBE+GlcCer combined treatment and CBE or GlcCer treatment.

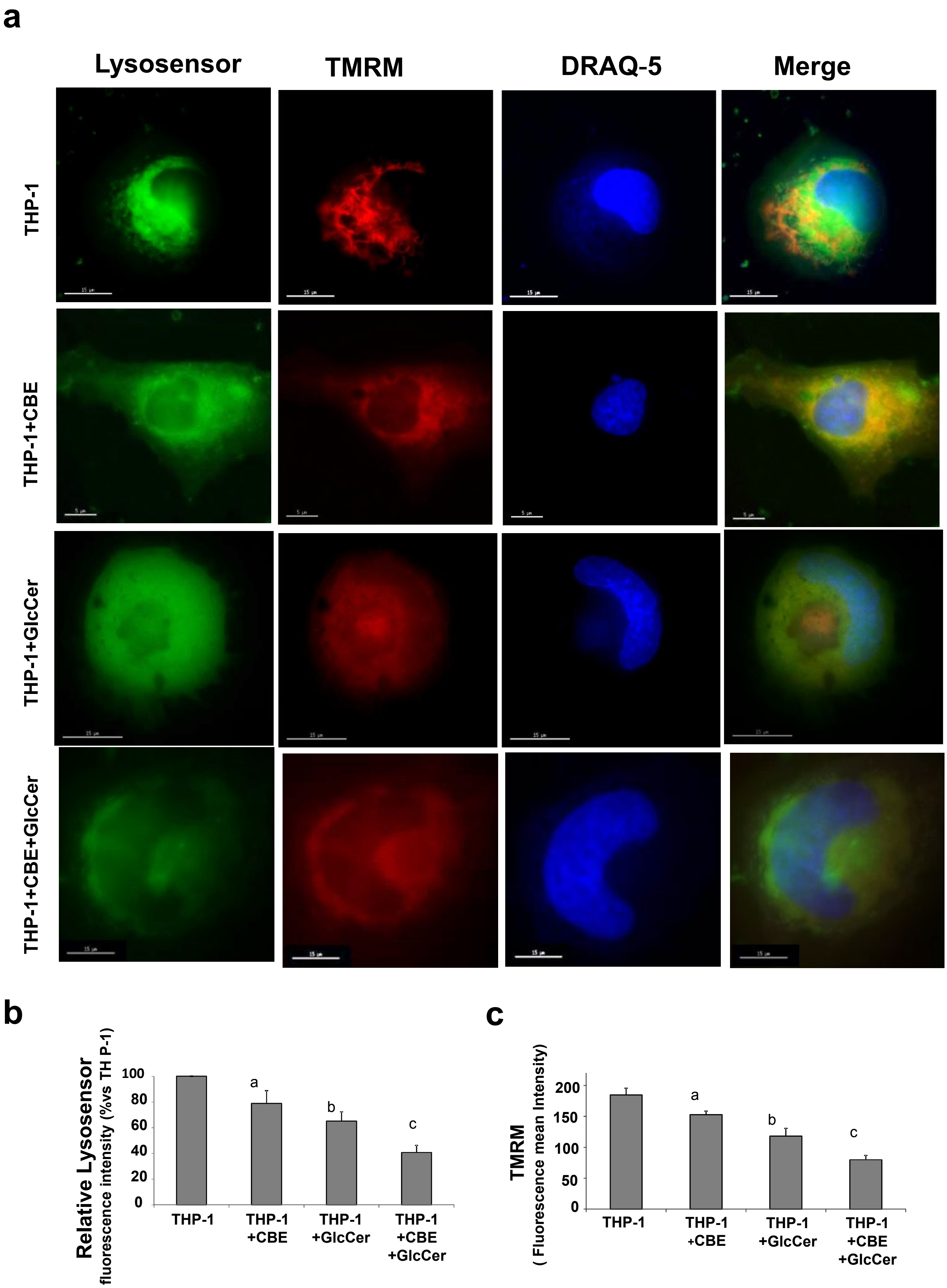
a
Lysosensor
TMRM
DRAQ-5
Merge
THP-1
THP-1+CBE
THP-1+GlcCer
THP-1+CBE+GlcCer
b
Relative Lysosensor
fluorescence intensity (%vs TH P-1)
100
80
60
40
20
0
a
b
c
THP-1
THP-1 +CBE
THP-1 +GlcCer
THP-1 +CBE +GlcCer
c
TMRM
(Fluorescence mean Intensity)
200
150
100
50
0
a
b
c
THP-1
THP-1 +CBE
THP-1 +GlcCer
THP-1 +CBE +GlcCer

S1

a

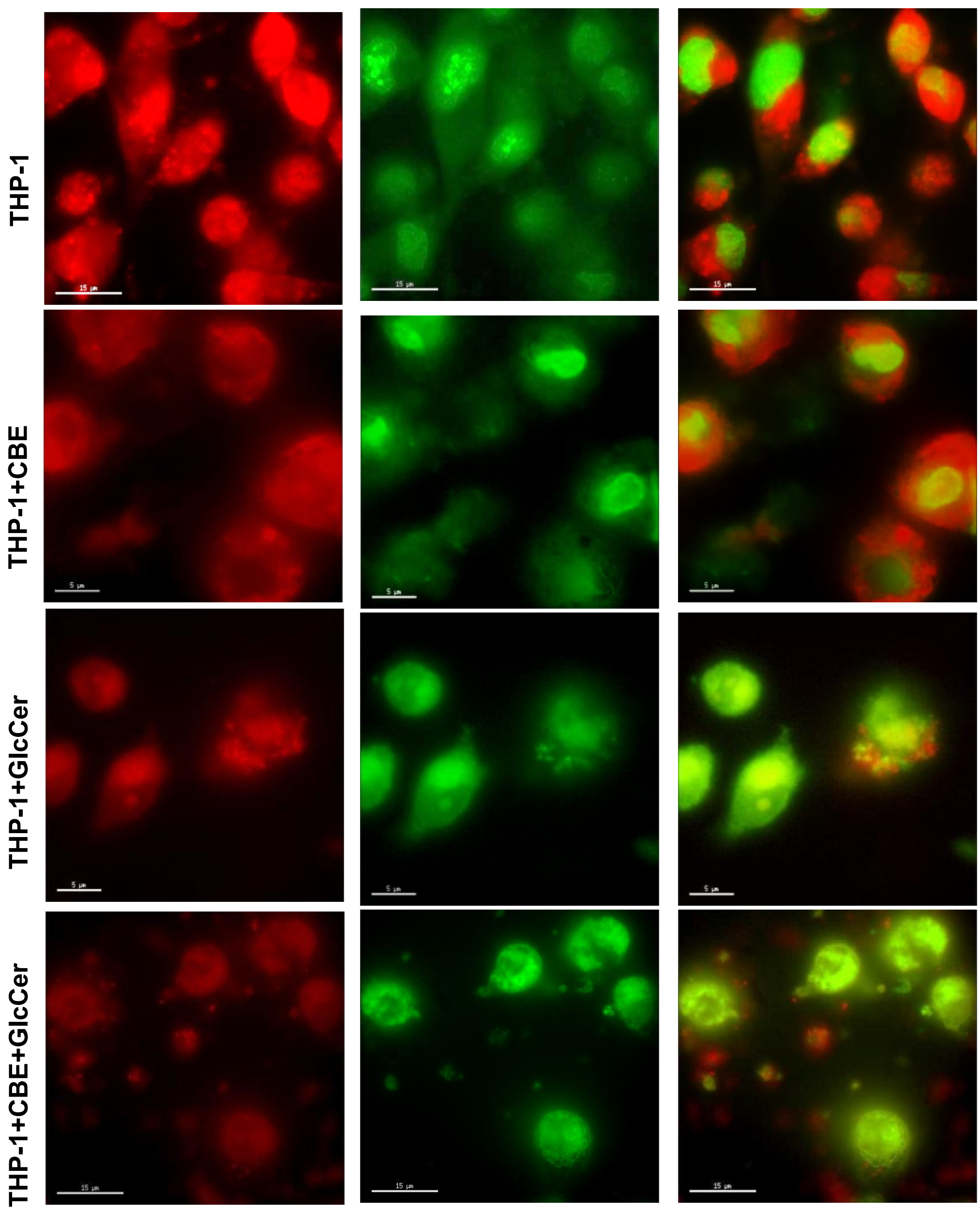
THP-1
THP-1+CBE
THP-1+GlcCer
THP-1+CBE+GlcCer

b

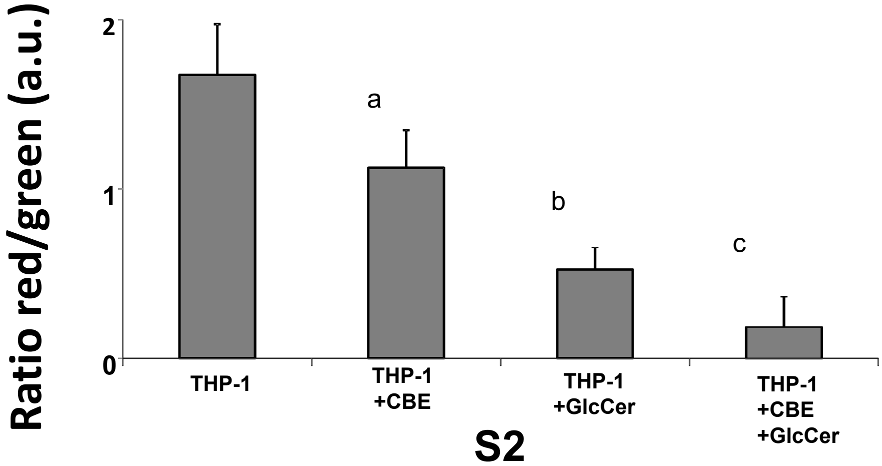
Ratio red/green (a.u.)
2
1
0
a
b
c
THP-1
THP-1
+CBE
THP-1
+GlcCer
THP-1
+CBE
+GlcCer

S2

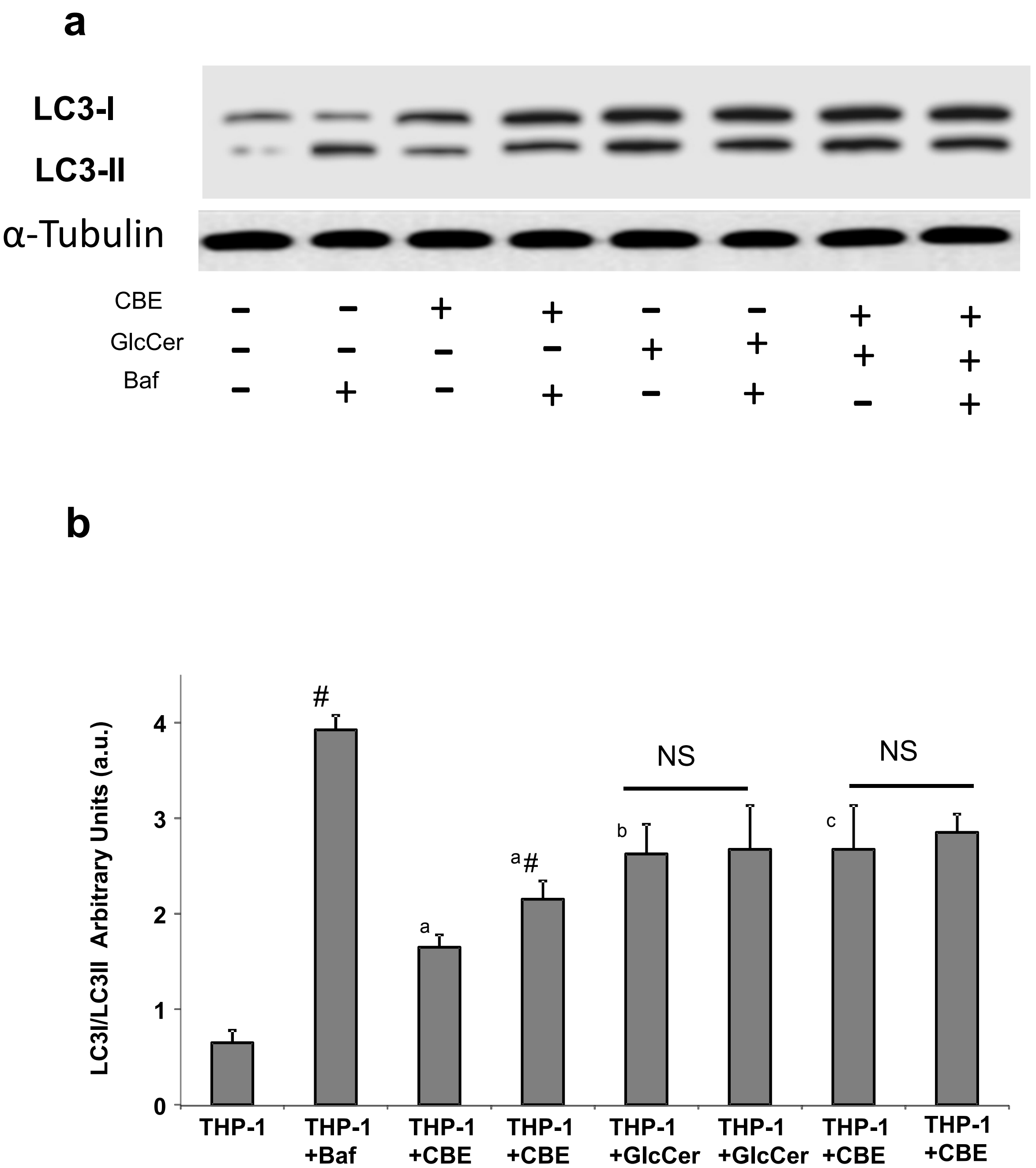
a
LC3-I
LC3-II
α-Tubulin
CBE
GlcCer
Baf
b
LC3I/LC3II Arbitrary Units (a.u.)
0
1
2
3
4
#
a
a#
b
c
NS
NS
THP-1
THP-1 +Baf
THP-1 +CBE
THP-1 +CBE +Baf
THP-1 +GlcCer
THP-1 +GlcCer +Baf
THP-1 +CBE +GlcCer
THP-1 +CBE +GlcCer +Baf

S3

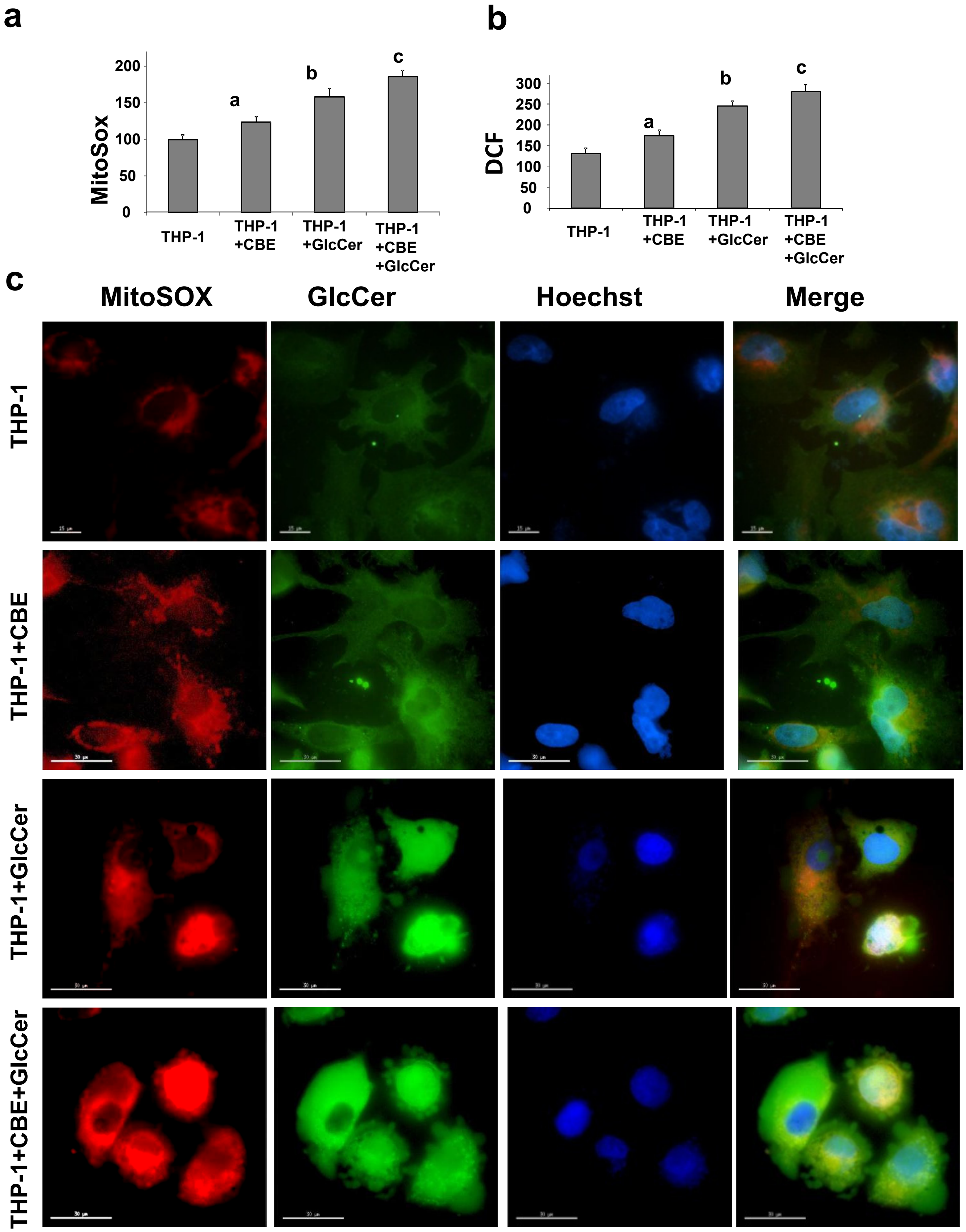
a
MitoSox
200
150
100
50
0
a
b
c
THP-1
THP-1 +CBE
THP-1 +GlcCer
THP-1 +CBE +GlcCer
b
DCF
300
250
200
150
100
50
0
a
b
c
THP-1
THP-1 +CBE
THP-1 +GlcCer
THP-1 +CBE +GlcCer
c
MitoSOX
GlcCer
Hoechst
Merge
THP-1
THP-1+CBE
THP-1+GlcCer
THP-1+CBE+GlcCer

S4

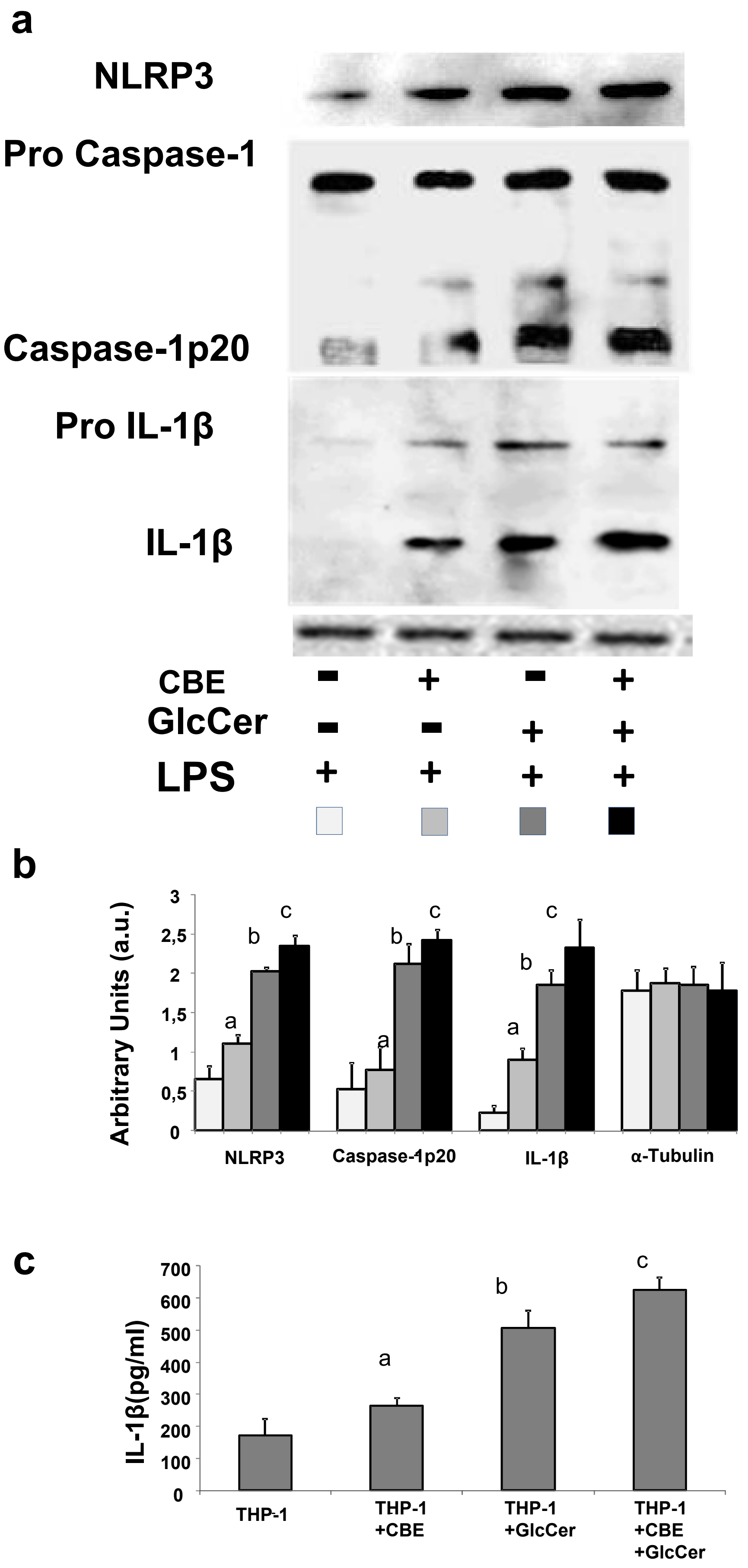
a
NLRP3
Pro Caspase-1
Caspase-1p20
Pro IL-1β
IL-1β
CBE
GlcCer
LPS
b
Arbitrary Units (a.u.)
3
2,5
2
1,5
1
0,5
0
a
b
c
NLRP3
Caspase-1p20
IL-1β
α-Tubulin
c
IL-1β(pg/ml)
700
600
500
400
300
200
100
0
THP-1
THP-1
+CBE
THP-1
+GlcCer
THP-1
+CBE
+GlcCer

S5

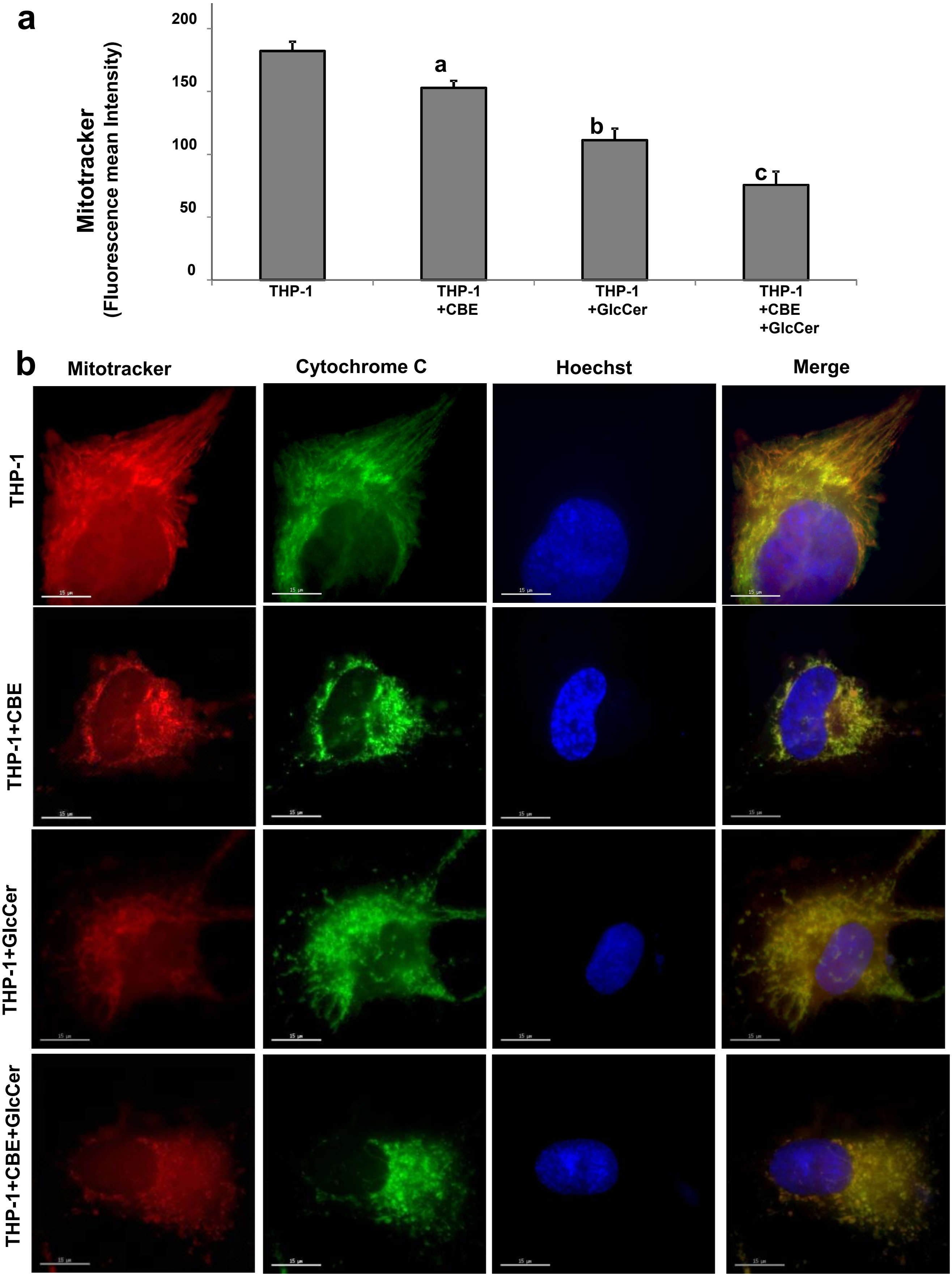
a
Mitotracker
(Fluorescence mean Intensity)
200
150
100
50
0
a
b
c
THP-1
THP-1
+CBE
THP-1
+GlcCer
THP-1
+CBE
+GlcCer
b
Mitotracker
Cytochrome C
Hoechst
Merge
THP-1
THP-1+CBE
THP-1+GlcCer
THP-1+CBE+GlcCer

S6

a

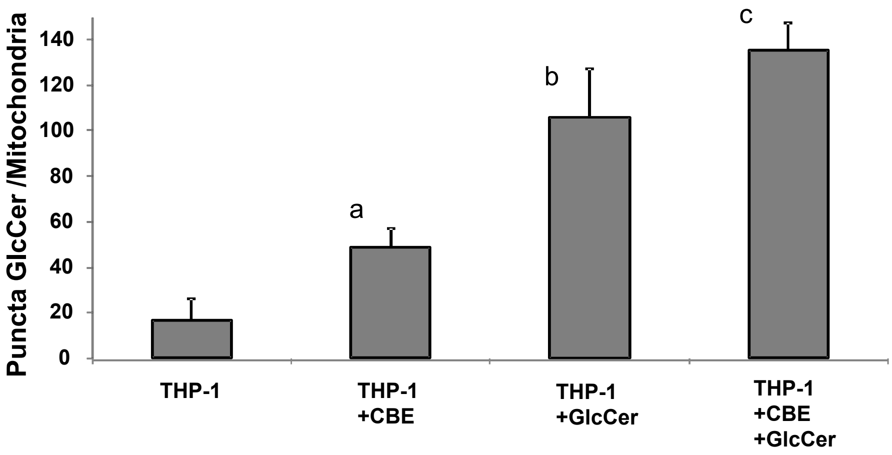
Puncta GlcCer /Mitochondria
0
20
40
60
80
100
120
140
a
b
c
THP-1
THP-1
+CBE
THP-1
+GlcCer
THP-1
+CBE
+GlcCer

b

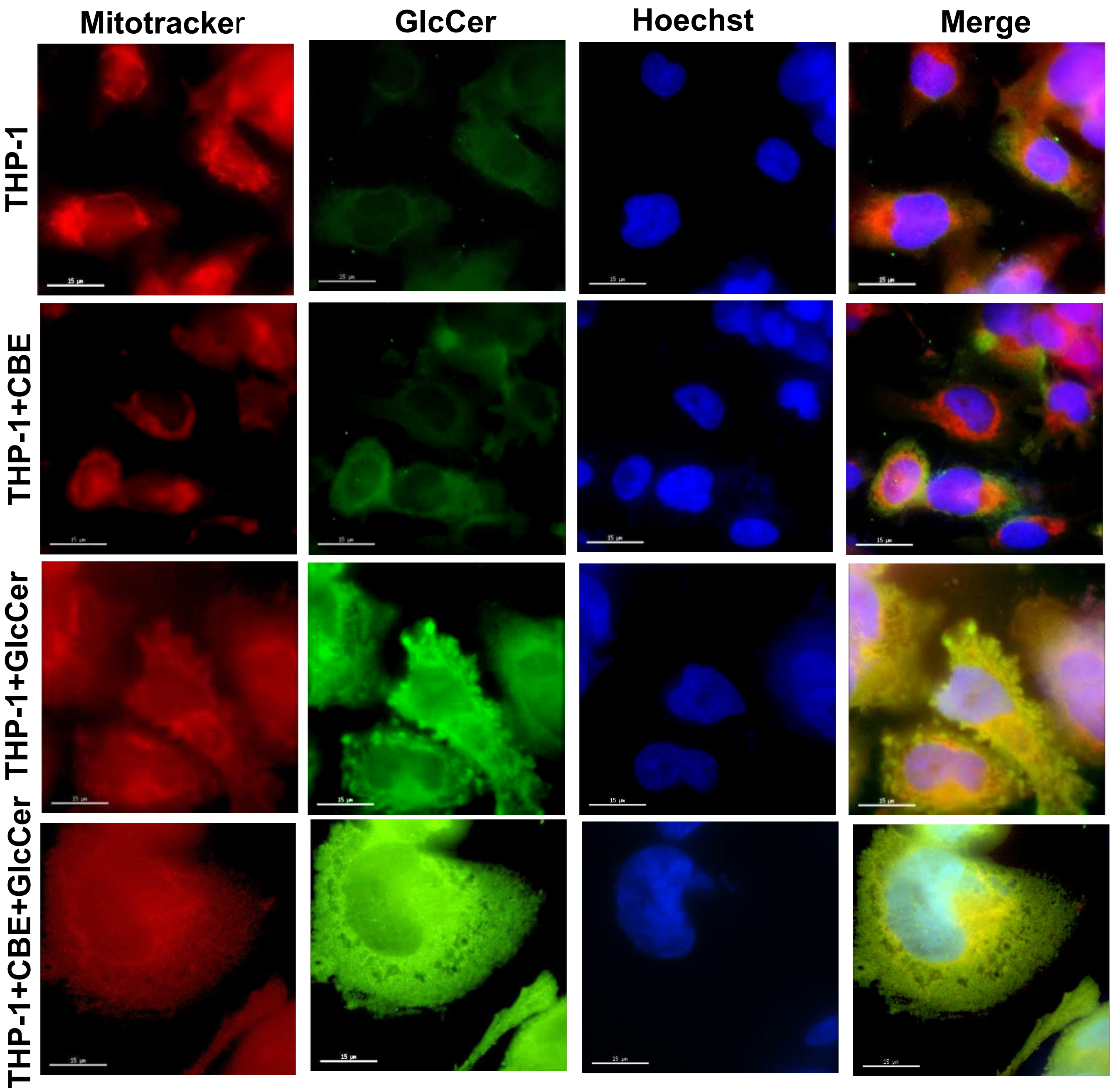
Mitotracker
GlcCer
Hoechst
Merge
THP-1
THP-1+CBE
THP-1+GlcCer
THP-1+CBE+GlcCer

S7

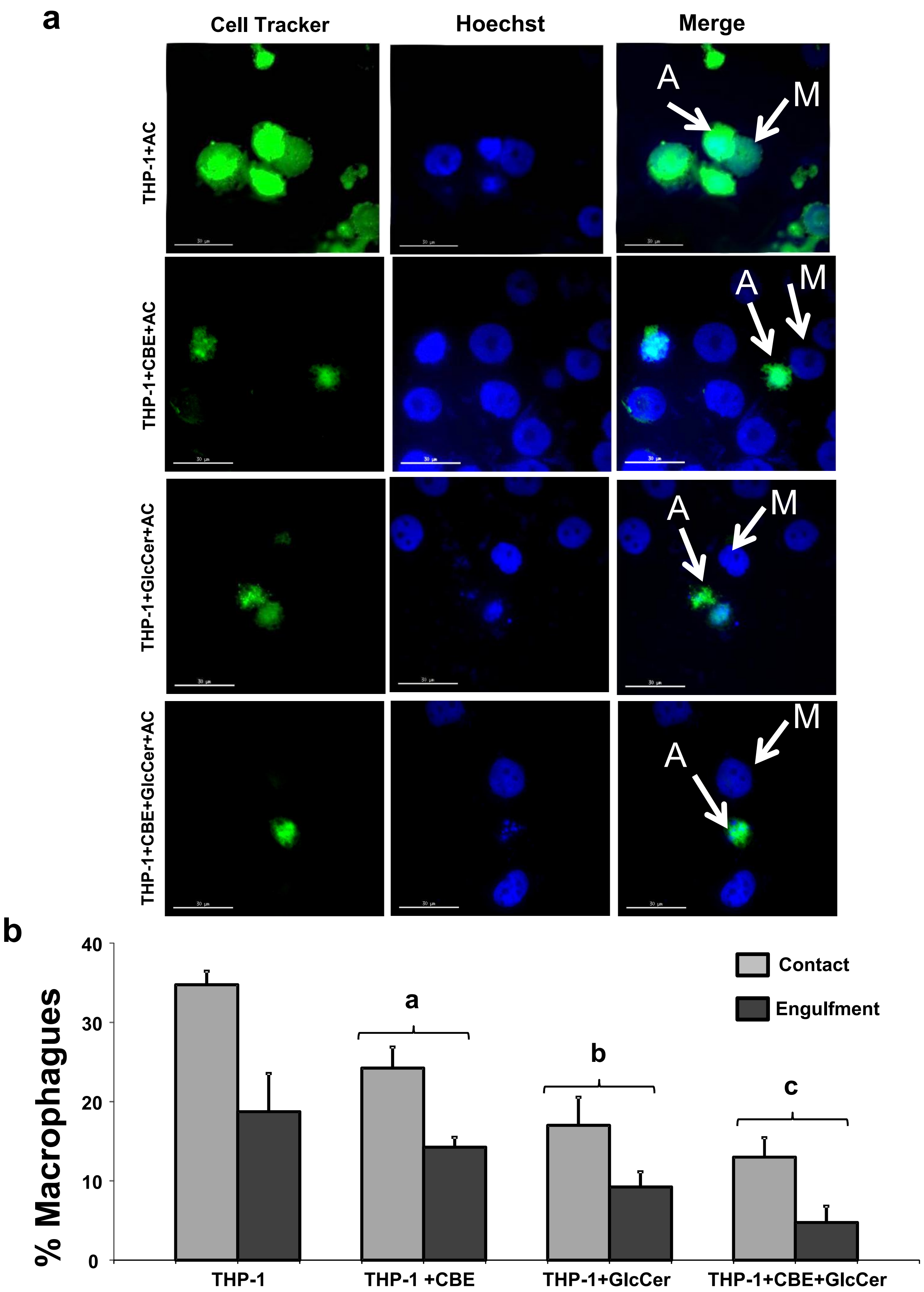
a
Cell Tracker
Hoechst
Merge
THP-1+AC
THP-1+CBE+AC
THP-1+GlcCer+AC
THP-1+CBE+GlcCer+AC
A
M
b
% Macrophages
40
30
20
10
0
Contact
Engulfment
a
b
c
THP-1
THP-1 +CBE
THP-1+GlcCer
THP-1+CBE+GlcCer

S8
